# The impact of the SARS-CoV-2 pandemic-related lockdowns on orthopedic trauma emergencies at a level-one trauma center

**DOI:** 10.1007/s00402-023-04947-2

**Published:** 2023-06-21

**Authors:** Valentin Messler, Tim Leschinger, Nadine Ott, Valentin Rausch, Volker Burst, Peer Eysel, Lars Peter Müller, Michael Hackl

**Affiliations:** 1 Faculty of Medicine, University Hospital Cologne, Center of Orthopedic and Trauma Surgery, and University of Cologne, Cologne, Germany; 2 Emergency Department, University of Cologne, Faculty of Medicine and University Hospital Cologne, Cologne, Germany; 3 Department II of Internal Medicine and Center for Molecular Medicine Cologne, University of Cologne, Faculty of Medicine and University Hospital Cologne, Cologne, Germany

**Keywords:** SARS-CoV-2, COVID-19, Lockdown, Orthopedics, Trauma, Emergency

## Abstract

**Introduction:**

The SARS-CoV-2 pandemic and its associated lockdowns had a profound effect on orthopedic trauma emergencies. This study aimed to investigate the patient volume and injury patterns at a level-one trauma center during the SARS-CoV-2 pandemic and compare them to the pre-pandemic conditions.

**Materials and methods:**

A retrospective chart review of all patients who presented to the orthopedic trauma emergency department of a level-one trauma center in Cologne, Germany within a 2 year period from March 16th, 2019 to March 15th, 2020 (pre-pandemic control) and from March 16th, 2020 and March 15th, 2021 (pandemic) was performed. The pandemic year was separated into three periods: (1) first lockdown, (2) between lockdowns and (3) second lockdown. The absolute numbers of patient presentations, the Manchester triage score (MTS) and the relative proportion of patients with structural organ injuries, fractures and dislocations, of polytraumatized patients, of hospital admissions, of subsequent emergency or semi-elective surgeries and of work-related accidents were evaluated in comparison to the pre-pandemic control.

**Results:**

A total of 21,642 patient presentations were included in this study. Significantly less weekly orthopedic trauma emergency patient presentations were recorded during the pandemic (*p* < 0.01).

The MTS was significantly lower during the first lockdown and between lockdowns (*p* < 0.01). The proportional incidence of overall structural organ injuries, fractures and dislocations, of upper limb fractures/dislocations, of hospital admissions and of patients requiring surgery was significantly increased during the pandemic (*p* ≤ 0.03). The proportional incidence of work-related injuries was significantly decreased during the pandemic (*p* < 0.01).

**Conclusions:**

Orthopedic trauma emergency presentations were reduced during the SARS-CoV-2 pandemic. Due to the reluctancy of patients to visit the emergency department during the pandemic, the proportions of relevant injuries in general and of upper limb injuries in particular as well as of patients requiring hospital admission and trauma-related surgery were significantly increased.

## Introduction

The emergence of the SARS-CoV-2 pandemic had a pronounced impact on society as a whole and on health care services in particular. To decelerate infection rates and to avoid exhaustion of medical resources and capacities, the German government employed a nationwide “lockdown” with closure of schools, daycares and all non-essential institutions and with restriction of social contacts. The first lockdown started on March 16th, 2020 and lasted approximately until May 11th, 2020 [1]. A majority of restrictions were subsequently lifted but were re-introduced on November 2nd, 2020 due to increasing infection rates [2]. Loosening of the restrictive measures of the second lockdown started on March 3rd, 2021 with social life slowly returning to pre-pandemic conditions [3].

The pandemic and its associated lockdowns with temporary restriction of everyday activities had a profound effect on orthopedic trauma emergency departments. Various international research was able to show that the lockdown policies led to an overall reduction in orthopedic trauma-related emergency presentations, hospital admissions and surgeries at the early stages of the pandemic [4–10]. Additionally, a relative shift towards low energy falls and domestic injuries was observed during the SARS-CoV-2 pandemic while motor vehicle accidents and sports-related injuries have reportedly decreased [10, 11]. Extensive analysis of injury patterns and their evolution over the course of the pandemic is lacking thus far.

Hence, this study aimed to investigate the patient volume and injury patterns at a level-one trauma center during the entire first year of the SARS-CoV-2 pandemic and compare them to the pre-pandemic conditions. We hypothesized that patient volumes would be decreased during the pandemic. Moreover, we hypothesized that the proportion of emergency trauma patients with significant injuries was higher during the pandemic when compared to the pre-pandemic conditions.

## Materials and methods

This investigation was approved by the institutional review board of the Medical Faculty of the University of Cologne (Approval number: 21-1153).

A retrospective chart review of all patients who presented to the orthopedic trauma emergency department of a level-one trauma center (Department of Orthopedic and Trauma Surgery of the University Hospital of Cologne) within a 2 year period from March 16th, 2019 to March 15th, 2021 was performed. The period from March 16th, 2019 to March 15th, 2020 was considered the control period with pre-pandemic conditions while the period from March 16th, 2020 to March 15th, 2021 represented the pandemic time frame.

### Data extraction

Age, sex, date of presentation and the Manchester triage score (MTS) upon arrival were obtained. Structural organ injuries, fractures and dislocations were extracted and classified as head/brain injuries (intracranial hemorrhage, skull or facial fractures), spine fractures, thoracic/abdominal injuries (rib fractures, sternum fractures, pneumothorax, hemothorax, pericardial effusion, structural injuries to the heart or the surrounding vessels; structural damage to internal organs), pelvic ring fractures, upper limb fractures and/or dislocations and lower limb fractures and/or dislocations. In addition to that, data regarding polytraumatized patients, hospital admissions, subsequent emergency or semi-elective surgeries and work-related accidents were extracted.

### Statistical analysis

The time frame from March 16th, 2020 to May 11th, 2020 was considered the first lockdown period, the time frame from May 12th, 2020 to November 1st, 2020 was considered the period between lockdowns and the period from November 2nd, 2020 to March 3rd, 2021 was considered the second lockdown period. The aforementioned data of these three time periods were compared to the data of the respective pre-pandemic control periods from March 16th, 2019 to March 3rd, 2020.

Normal distribution of ordinal and numerical variables was analyzed with a Kolmogorow–Smirnow test. An independent *t* test was performed to evaluate significant differences regarding the number of weekly presentations; homogeneity of variance was verified with a Levene test. The MTS was analyzed using a Mann–Whitney *U* test. Binary variables were evaluated using Fisher’s exact test. The level of significance was set at *p* < 0.05.

## Results

A total of 21,642 patient presentations were included in this study. The mean age of patients was 32 years (± 23 years). 55.0% of patients were male and 45.0% were female.

**Fig. 1:**
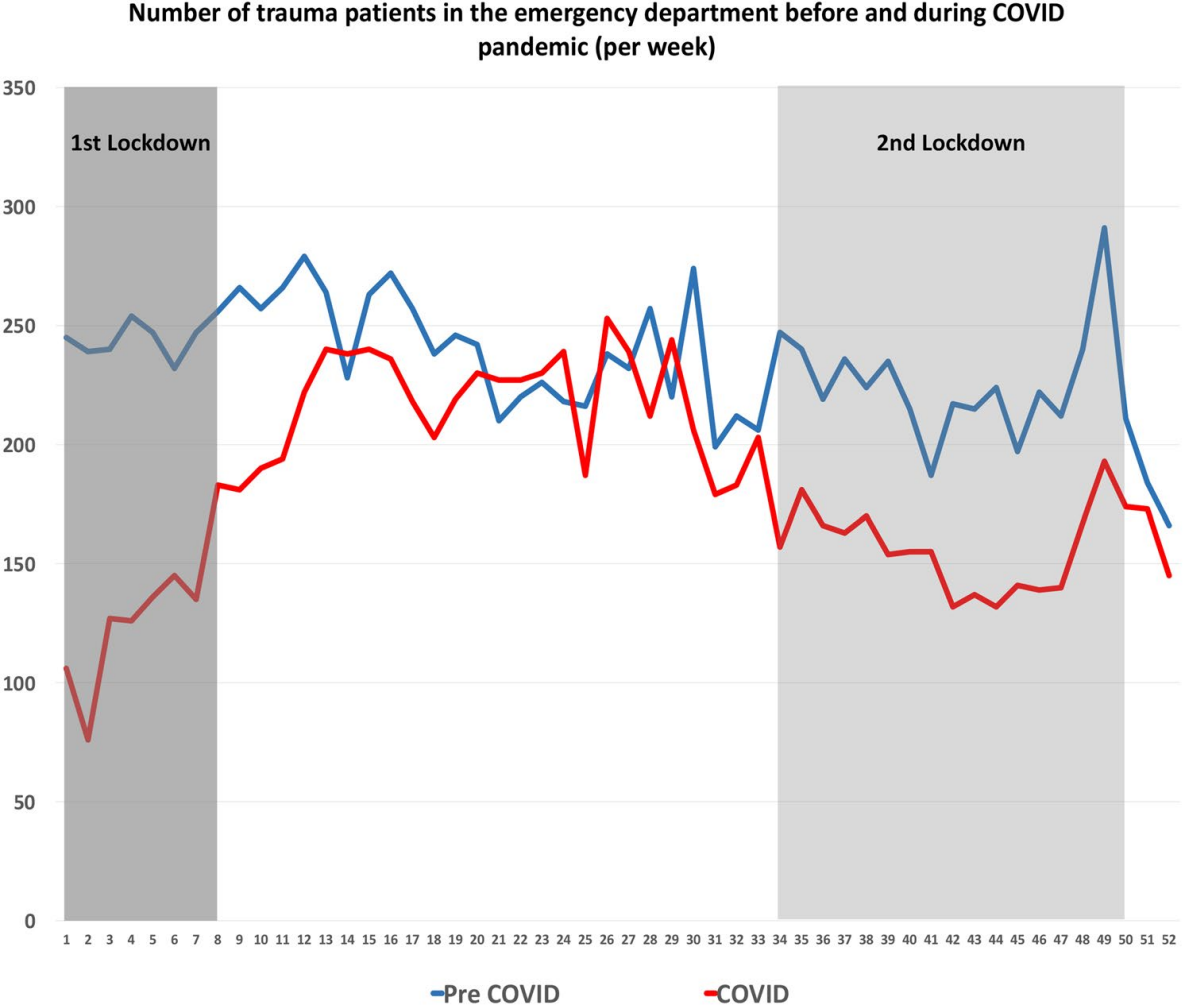
Emergency trauma patient presentations during the pandemic (March 16th, 2020–March 15th, 2021; red line) were significantly lower when compared to the control period before the pandemic (March 16th, 2019–March 15th, 2020; blue line). The lockdown periods are marked in gray

During the first lockdown period, between lockdowns and during the second lockdown, significantly less weekly orthopedic trauma emergency patient presentations were recorded when compared to the control period (*p* < 0.01) (Table 1, Fig. 1).

**Table 1:** Comparison of orthopedic trauma emergency patient presentations per week during the first lockdown, between lockdowns and during the second lockdown

Patient presentations per week	Mean	SD	*p* value
First lockdown	135	34	< 0.01
Control period	247	10	
Between lockdowns	219	21	< 0.01
Control period	242	23	
Second lockdown	156	18	< 0.01
Control period	225	23	

All values are significantly lower when compared to the pre-pandemic control periods

*SD* standard deviation

The Manchester Triage Score was significantly lower during the first lockdown (mean rank 1644 versus 1763; *p* < 0.01) and between lockdowns (mean rank 5396 versus 5595; *p* < 0.01) when compared to the control periods. During the second lockdown, no significant differences were observed (mean rank 3265 versus 3231; *p* = 0.42).

**Fig. 2:**
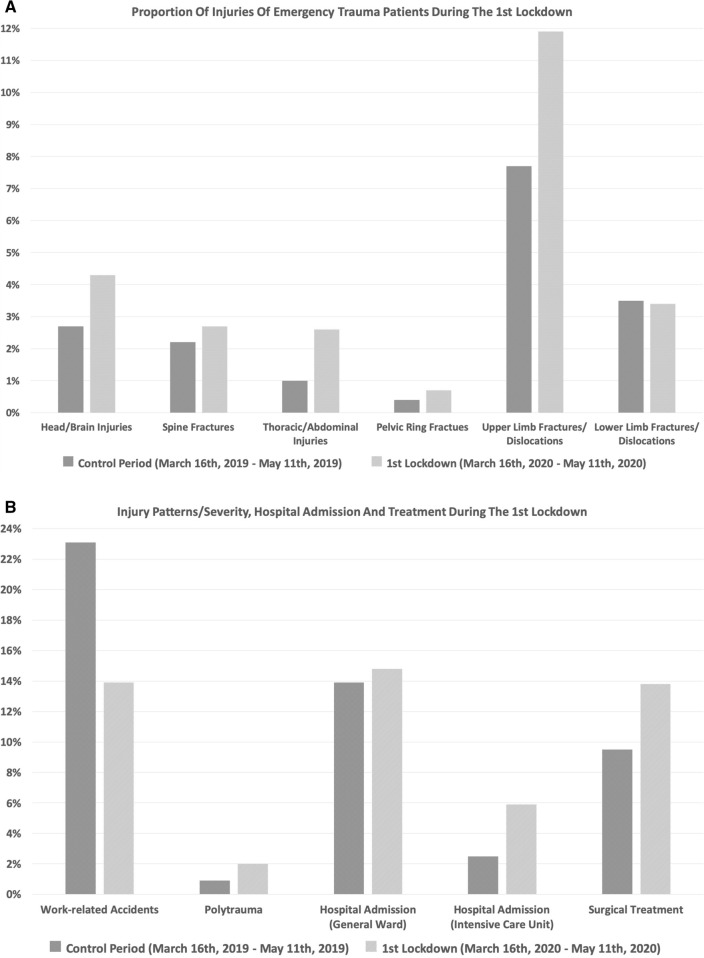
**A**, **B** Proportional incidence of injuries and trauma characteristics during the first lockdown compared to the control period. The proportional incidence of head and brain injuries (*p* = 0.03), thoracic/abdominal injuries (*p* < 0.01), upper limb fractures/dislocations (*p* < 0.01), polytrauma patients (*p* = 0.01), hospital admissions (*p* < 0.01) and patients requiring surgical treatment (*p* < 0.01) was significantly increased. The proportional incidence of work-related accidents was significantly decreased (*p* < 0.01)

During the first lockdown, the proportional incidence—in relation to total patient presentations—of overall structural organ injuries, fractures and dislocations was significantly increased (*p* < 0.01). Likewise, the proportional incidence of head and brain injuries (*p* = 0.03), thoracic/abdominal injuries (*p* < 0.01), upper limb fractures/dislocations (*p* < 0.01), polytrauma patients (*p* = 0.01), hospital admissions (*p* < 0.01) and patients requiring surgical treatment (*p* < 0.01) was significantly increased. The proportional incidence of work-related injuries was significantly decreased during the first lockdown (*p* < 0.01). No significant differences in the proportional incidence of spine fractures (*p* = 0.35), pelvic ring fractures (*p* = 0.29) and lower limb fractures/dislocations (*p* = 0.92) were observed (Fig. 2A, B).

**Fig. 3:**
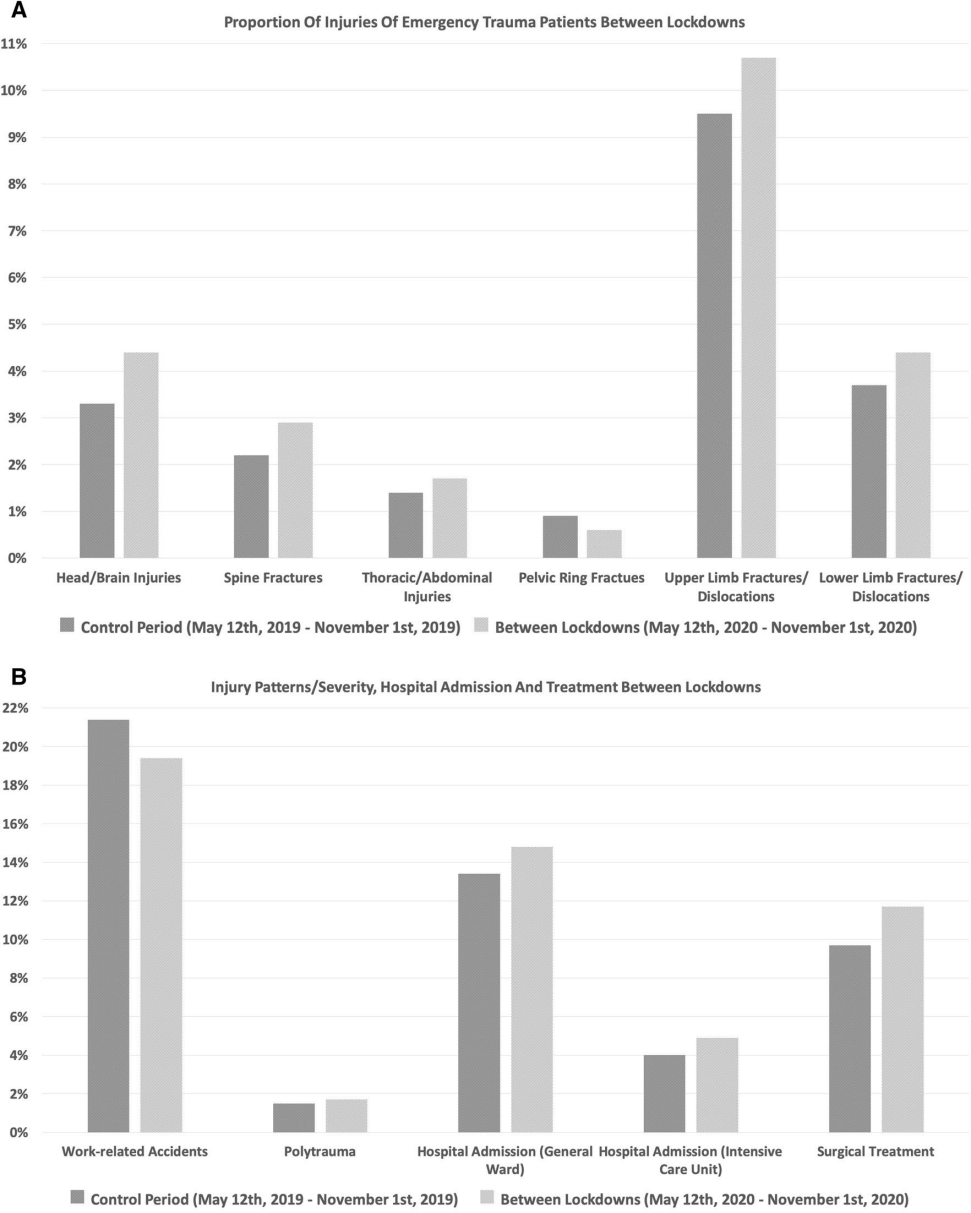
**A**, **B** Proportional incidence of injuries and trauma characteristics between lockdowns compared to the control period. The proportional incidence of head and brain injuries (*p* < 0.01), spine fractures (*p* = 0.04), upper limb fractures/dislocations (*p* = 0.03), hospital admissions (*p* < 0.01) and patients requiring surgical treatment (*p* < 0.01) was significantly increased. The proportional incidence of work-related injuries was significantly decreased (*p* = 0.01)

Between lockdowns, the proportional incidence of overall structural organ injuries, fractures and dislocations (*p* < 0.01), head and brain injuries (*p* < 0.01), spine fractures (*p* = 0.04), upper limb fractures/dislocations (*p* = 0.03), hospital admissions (*p* < 0.01) and patients requiring surgical treatment (*p* < 0.01) was significantly increased. The proportional incidence of work-related injuries was significantly decreased between lockdowns (*p* = 0.01). No significant differences in the proportional incidence of thoracic/abdominal injuries (*p* = 0.22), pelvic ring fractures (*p* = 0.08), lower limb fractures/dislocations (*p* = 0.08) and polytrauma patients (*p* = 0.32) were observed (Fig. 3A, B).

**Fig. 4:**
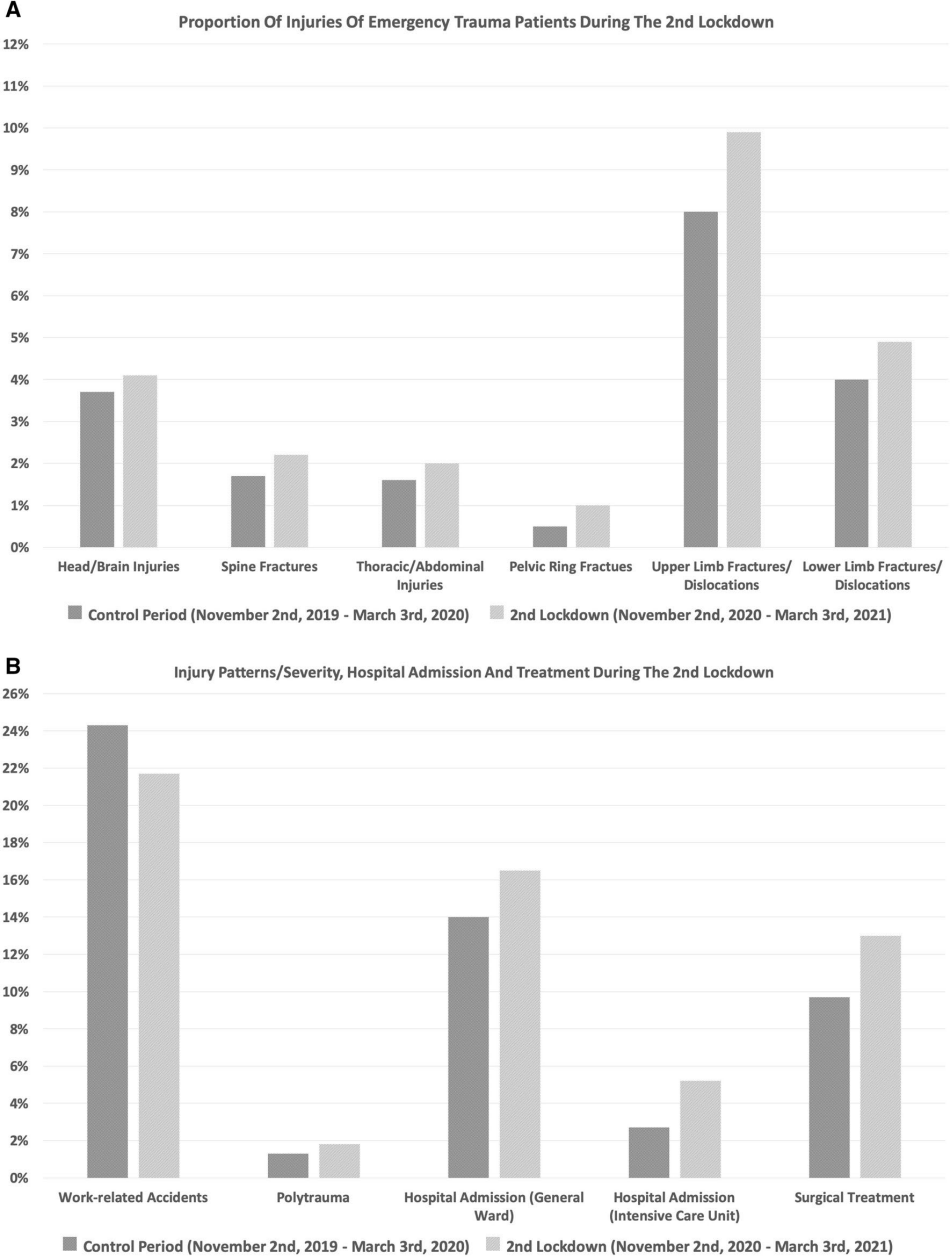
**A**, **B** Proportional incidence of injuries and trauma characteristics during the second lockdown compared to the control period. The proportional incidence of pelvic ring fractures (*p* = 0.02), upper limb fractures/dislocations (*p* = 0.01), hospital admissions (*p* < 0.01) and patients requiring surgical treatment (*p* < 0.01) was significantly increased. The proportional incidence of work-related injuries was significantly decreased (*p* = 0.02)

During the second lockdown, a significant increase of the proportional incidence of overall structural organ injuries, fractures and dislocations (*p* < 0.01), pelvic ring fractures (*p* = 0.02), upper limb fractures/dislocations (*p* = 0.01), hospital admissions (*p* < 0.01) and patients requiring surgical treatment (*p* < 0.01) was seen. The proportional incidence of work-related injuries was significantly decreased during the second lockdown (*p* = 0.02). No significant differences in the proportional incidence of head and brain injuries (*p* = 0.44), spine fractures (*p* = 0.17), thoracic/abdominal injuries (*p* = 0.29), lower limb fractures/dislocations (*p* = 0.10) and polytrauma patients (*p* = 0.19) were observed (Fig. 4A, B).

## Discussion

This study aimed to investigate the patient volume and injury patterns at a level-one trauma center during the SARS-CoV-2 pandemic and compare them to the pre-pandemic conditions. Our results confirm that overall orthopedic trauma emergency patient presentations were reduced during the SARS-CoV-2 pandemic, especially during the two lockdown periods. More importantly, we were able to show that the proportion of relevant injuries in general and of upper limb injuries as well as of patients requiring hospital admission and surgery were significantly higher during the pandemic. The proportion of work-related accidents on the other hand decreased significantly throughout the pandemic. These effects seemed to be more pronounced during the first lockdown period.

The reduction of work-related injuries can partly be attributed to the fact that more people were working remotely and that many facilities were temporarily closed. This correlates to the findings of other authors. Staunton et al. reported a significant decrease in sports-related injuries in Ireland during the first lockdown period and Hakkenbrak et al. as well as Moyer et al. found less motor vehicle accidents in the Netherlands and in France during the first two months of the SARS-CoV-2 pandemic [10, 12].

While Gumina et al. observed an absolute reduction in shoulder and elbow injuries in Italy during the first month of the pandemic [13], we found a relative increase of upper extremity fractures and dislocations when compared to the total number of patients throughout the first year of the SARS-CoV-2 pandemic. Our findings correspond well to those of the aforementioned authors from the Netherlands who also reported a relative increase of upper extremity injuries but—in contrast to our investigation—also of lower extremity injuries [12].

Some authors reported a reduction of high energy trauma and a relative increase of low energy falls, particularly in elderly patients [4, 11]. More precisely, the proportion of domestic accidents rose to 60% in February and March of 2020 compared to just 14% the year prior according to the study of Pogetti et al. [11]. Additionally, 56% of their patients were older than 50 years in February and March of 2020 while in 2019 this age group made up for only 26% of all emergency trauma presentations [11]. The reduction of high velocity trauma due to less traffic and closure of public facilities and the relative increase of domestic falls—which were not reduced as a result of the lockdown measures—may help to explain why we found significantly higher proportions of isolated upper limb injuries throughout the first year of the pandemic. This development is also consistent with the findings of several studies that hip fractures in elderly patients were not relevantly reduced during the SARS-CoV-2 pandemic [6, 9, 14–16]. Murphy et al. reported that not only proximal femur fractures but also periprosthetic hip fractures and prosthetic hip joint dislocations remained at a similar frequency during the SARS-CoV-2 pandemic when compared to the 3 years prior [9].

The results of our investigation regarding a relative increase in relevant injuries, patients requiring emergency or semi-elective surgery and hospital admissions are probably a direct result of patients being more reluctant to visit the emergency department for minor injuries during the pandemic and these data are supported by other authors [17–20]. Kreis et al. reported that the relative incidence proportions of emergency trauma surgeries in their level-one trauma center in Germany increased from 8.8% before the pandemic to 12.2% during the first month of the pandemic [19]. In our study cohort, emergency and semi-elective surgeries as a result of emergency trauma presentations were performed in 9.7% of patients prior to the pandemic and in 12.3% during the first year of the SARS-CoV-2 pandemic. According to the Manchester Triage Score, a larger proportion of our patients were presenting to our level-one trauma center with potentially serious injuries during the pandemic. This corresponds well to the findings of Esteban et al. who found a relative increase in emergency visits (triage levels 1 to 3 of 5) in their orthopedic trauma emergency department in Spain from 22% in 2018 and 2019 to 40% in 2020. Anwander et al. published a study from their level-one trauma center in Switzerland stating that the median Injury Severity Score was significantly higher (25 in 2020 versus 22 in 2019; *p* = 0.04) and that a larger percentage of patients were admitted to an intensive care unit during the pandemic. This data is supported by our findings as we have seen a higher proportion of hospital admissions during the entire first year of the pandemic and a significantly higher proportion of polytrauma patients during the first lockdown.

### Limitations

This study is limited by its retrospective and monocentric design. As a level-one trauma center, our results may not necessarily be representative for the national and international orthopedic trauma emergency landscape. Additionally, the control period of 1 year may not be representative and thus might present an additional confounding factor.

## Conclusions

The results of this investigation show that overall orthopedic trauma emergency patient presentations were reduced during the SARS-CoV-2 pandemic, especially during the two lockdown periods. This is most probably a direct result of the lockdown measures with reduction of social contacts and closure of public facilities. Moreover, patients seem to have been more reluctant to visit the emergency department for minor injuries. Therefore, we were able to show that the proportion of relevant injuries in general and of upper limb injuries in particular as well as of patients requiring hospital admission and surgery were significantly higher during the pandemic.
